# Coping strategy enhancement for the treatment of distressing voices in young people: A service evaluation within routine clinical practice

**DOI:** 10.1177/13591045211061803

**Published:** 2022-03-09

**Authors:** Mark Hayward, Hazel Frost, Akira Naito, Anna-Marie Jones

**Affiliations:** 1 R&D Department, Sussex Education Centre, 8944 Sussex Partnership NHS Foundation Trust, Hove, UK; 2 School of Psychology, 1948 University of Sussex, Brighton, UK

**Keywords:** Hearing voices, young people, coping, intervention, child & adolescent mental health service

## Abstract

**Background:**

Hearing voices is a common experience in young people, irrespective of diagnosis. This experience can be associated with distress, self-harm and an increased risk of attempting suicide. However, there are currently no evidence-based interventions which specifically target distressing voice hearing experiences in young people.

**Method:**

This was a service evaluation exploring the engagement, outcomes and experiences of young people who were offered a brief 4-session intervention for distressing voices within a Child and Adolescent Mental Health Service (CAMHS) in the UK’s National Health Service. The intervention was based on the principles of Coping Strategy Enhancement (CSE).

**Results:**

A total of 24 young people were offered the CSE intervention over a 20-month period. The intervention was completed by 15 young people. Pre-post outcomes suggested clinically meaningful reductions in the negative impact of voices for the majority of the young people. Qualitative feedback was positive and highlighted the value of both a space to talk about voice hearing experiences and a focus upon coping strategies.

**Conclusions:**

The findings from this service evaluation suggest that CSE can be a brief, acceptable and helpful way for young people within a CAMHS context to start a therapeutic conversation about their distressing voice hearing experiences.

## Introduction

Hearing a voice that no one else can hear can be a very distressing experience and is usually associated with adults who have been given a diagnosis of psychosis. However, it is becoming increasingly apparent that distressing voice hearing experiences are experienced by people of all ages who have a range of mental health problems (Maijer et al., 2019; Waters & Fernyhough, 2017). Somewhat surprisingly, voice hearing is most common amongst young people (Maijer et al., 2017), albeit this experience is likely to be transient (Bartels-Velthuis et al., 2016). Studies have found that voice hearing in young people can be associated with distress (Maijer et al., 2017), self-harm (Nishida et al., 2010) and an increased risk of attempting suicide (Kelleher et al., 2013).

Irrespective of the context in which it occurs, voice hearing seems to be difficult to talk about. This difficulty has been known for some time in the mental health services provided to adults (Coffey & Hewitt, 2008). We have explored this difficultly in the mental health services provided to younger people and our research has suggested that both younger people and clinicians can feel anxious about having a conversation about voice hearing experiences. Younger people can feel ashamed and stigmatised by their voice hearing experiences and can fear a negative response from the voices and/or the clinician if they discuss these experiences (Bogen-Johnston et al., 2019). Clinicians can have concerns about making things worse and being out-of-their-depth within this conversation (Bogen-Johnston et al., 2020). Recommendations for stimulating and supporting the initiation of these conversations include the use of structured assessment tools and training to enhance practitioner confidence (Bogen-Johnston et al., 2020).

If voice hearing experiences are discussed with young people and intervention is considered necessary, there are currently no evidence-based interventions available which specifically target distressing voices. Interventions informed by the principles of Cognitive Behaviour Therapy (CBT) are being developed and evaluated for a broader array of ‘unusual experiences’ (Jolley et al., 2018). In the context of the transient nature of voice hearing experiences for the majority of young people, a ‘curious but cautious’ approach to intervention is recommended (Maijer et al., 2019). The Stronger Than Your Voices intervention has recently been evaluated in the Netherlands and embraces this approach by using modules to gradually build the layers of the intervention (Maijer et al., 2020). Within the UK, we have developed a brief coping intervention for the distressing voices experienced by adults. This intervention draws upon the principles of Coping Strategy Enhancement (CSE - Tarrier et al., 1992) and uses a functional analytic framework to explore the triggers and responses to voices. Brief CSE has been found to be a helpful way to begin a conversation with adults about their voice hearing experiences (Hayward et al., 2018). We wondered whether this intervention might be a light-touch and helpful way to guide conversations about the voice hearing experiences of young people.

The Sussex Voices Clinic (SVC - https://www.sussexpartnership.nhs.uk/sussex-voices-clinic) is currently piloting the delivery of brief CSE to young voice hearers who are receiving a service from a Child and Adolescent Mental Health Service (CAMHS) in the UK’s National Health Service (NHS). This service evaluation reports the engagement, outcomes and experiences for a cohort of young people who were offered brief CSE within SVC.

## Method

### Design

This project was undertaken as a service evaluation exploring engagement, outcomes and experiences for young people referred from CAMHS to SVC. As this study was a service evaluation of routine clinical practice, NHS Research Ethics Committee approval was not required (UK Policy Framework for Health and Social Care Research; Department of Health, 2017). This service evaluation was registered with an NHS audit department (dated August 26, 2015) who advised that informed consent from young people was not necessary. All data in this evaluation have been anonymised.

SVC is a trans-diagnostic outpatient service offered to young people and adults within an NHS Mental Health Trust in Sussex, UK. SVC piloted its service within a single CAMHS team from March 2019 to December 2020. Referrals were made by CAMHS clinicians after discussions with the young person and the multi-disciplinary team. SVC screened the referrals and, if appropriate, offered a baseline assessment to the young person. The eligibility criteria required young people to score at least eight (range from 0–16) on the negative impact scale of the Hamilton Program for Schizophrenic Voices Questionnaire (HPSVQ; Van Lieshout & Goldberg, 2007). All young people who met the referral criteria were offered a 4-session course of brief CSE.

### Intervention

The brief CSE intervention consisted of a maximum of four 60-minute sessions (offered approximately weekly) of individual therapy, guided by a workbook (a copy of the workbook is available to download from https://www.sussexpartnership.nhs.uk/resources-sussex-voices-clinic). We consulted with CAMHS clinicians about the need for the intervention to be adapted for young people. There was agreement that the intervention may be suitable in its current form and changes should only be made in the light of experience. Consequently, the intervention was unchanged from the intervention delivered to adults and was offered as follows:

Session 1: a semi-structured interview (adapted from the Antecedent and Coping Interview; Tarrier et al., 1990) is used to identify the antecedents to voice activity (e.g. striving to achieve high standards, nighttime, feeling anxious/low in mood/angry, etc.) and the young person’s emotional and behavioural responses to the voices (self-harm, spending time with friends, taking back to voices, etc.). The ability of these responses to facilitate coping or increase distress is considered. These discussions stimulate a process of identifying coping strategies and evaluating their effectiveness.

Session 2: an existing coping strategy is collaboratively selected and considered in detail. Discussions focus on how the strategy could be modified and used differently (more or less often). A plan is agreed to implement the strategy between sessions.

Session 3: implementation of the modified coping strategy is reviewed. Discussions focus on the enablers and barriers to implementation, and the effectiveness of the strategy. This strategy could be further modified to enhance effectiveness, or another strategy could be selected and modified. A plan is agreed to implement the strategy between sessions.

Session 4: implementation of the modified coping strategy is reviewed, and any required modifications are agreed upon. Plans are discussed for continued implementation post-intervention. Discussions explore any learning from the intervention in relation to both self and voices, and the implications of this learning for living well with voices. Any needs for further intervention are discussed.

The intervention was delivered by clinicians with varying degrees of experience in delivering therapeutic interventions. There were 11 therapists in total with the following roles: clinical psychologist, trainee clinical psychologist, mental health nurse, art therapist, occupational therapist and assistant psychologist. Therapists were trained in the delivery of brief CSE during a 90-minute training session facilitated by the first author (a Clinical Psychologist with extensive experience of working with adults who are distressed by hearing voices) who also provided monthly supervision.

All young people were receiving treatment-as-usual from CAMHS during the course of the service evaluation.

### Assessment and measures

Young people were assessed by a clinic assistant not involved in delivering the intervention (in order to reduce risk of bias) at two time points: (1) baseline – within four weeks before starting CSE and (2) post-intervention – within four weeks of finishing CSE. Baseline assessments included the collection of demographic information and the following clinical outcomes:

### Primary clinical outcome

Hamilton Program for Schizophrenic Voices Questionnaire (HPSVQ) (Van Lieshout & Goldberg, 2007) is a 9-item self-report measure assessing physical characteristics of voices and negative impact of voices across two scales (Kim et al., 2010). The two-factor structure of the HPSVQ has recently been corroborated (Berry et al., 2021). The four items of the negative impact of voices scale were as follows: Q2, How bad are the things the voices say to you? Q5, How much do the voices interfere with your daily activities? Q6, How distressing are the voices that you hear? and Q7, How bad (worthless/useless) do the voices make you feel about yourself? Each item is scored on a scale from 0 (least severe or impairing) to 4 (most severe or impairing), generating a range of scores from 0–16.

### Secondary clinical outcomes

 Physical characteristics of voices scale is a 5-item measure within the HPSVQ (Van Lieshout & Goldberg, 2007). Each item is scored on a scale from 0 to 4, generating a range of scores from 0-20.

Choice of Outcome in CBT for Psychoses (CHOICE-SF) is a 12-item shortened version of Greenwood et al.’s (2010) self-report questionnaire assessing service user goals for CBT for psychosis that are relevant to subjective recovery. Items are rated on a 0–10 scale (0 = worst and 10 = best). The first eleven items are rated to create a severity score and an optional twelfth item is a free text item where respondents can insert their personal goal. The psychometric properties of the short form have recently been evaluated and results demonstrated high internal consistency, high levels of sensitivity to change and good construct validity (Webb et al., 2020).

Patient Health Questionnaire (PHQ-9) is a 9-item self-report measure of depression symptom severity. Items are rated on a 4-point scale. Scores under 10 are considered sub-clinical. The scale has good levels of sensitivity and specificity (Gilbody et al., 2007). Although the optimal cut point is higher among adolescents, the sensitivity and specificity of the PHQ-9 are similar to those of adult populations (Richardson et al., 2010).

Generalised Anxiety Disorder 7 (GAD-7) is a seven-item measure of generalised anxiety. Items are rated on a 4-point scale. Scores represent: 5-9 mild; 10-14 moderate; 15-21 severe anxiety. GAD-7 has excellent psychometric properties (Spitzer et al., 2006). The psychometric properties of the GAD-7 in adolescents have been found to be similar to those reported among adults (Tiirikainen et al., 2019).

### Qualitative feedback

At the post-intervention assessment, young people were invited to offer comments on their experience of receiving brief CSE within SVC.

### Statistical analysis

Descriptive statistics [count (N), mean (M), standard deviation (*SD*), min, max] were used to summarise the demographics of the young people (age, gender, ethnicity, education, age at voice hearing onset and duration of voice hearing).

When interpreting the differences in the primary and secondary outcomes, the minimal clinically important difference (MCID) was used as a reference point to determine whether differences between outcomes at baseline and post-intervention were considered meaningful. Percentages of those who had improved or deteriorated by at least the MCID were calculated. The relevant MCIDs were 2 points on the HPSVQ negative impact of voices scale (Hazell et al., 2018) and 1.45 on the CHOICE-SF (Jolley et al., 2015). Standardised effect sizes (Cohen’s d) were calculated as the mean pre-post difference divided by the baseline *SD* taking into account the pre and post-measurement correlation. Standardised effects were interpreted in line with Cohen’s criteria (Cohen, 1960, that is, small = .2, medium = .5 and a large effect = .8). 95% CIs were calculated for all estimates. Missing data was assessed but no attempts were made to replace it. All analyses were carried out using SPSS V24.

## Results

Between March 2019 and December 2020, 24 young people were referred to SVC, assessed as eligible for and offered the brief CSE intervention. Most of the young people were female; they ranged in age from 12–17 years, had been hearing voices for a mean of 3 years and were representative of the geographical region with regard to ethnicity. The young people had been receiving a service from CAMHS for between 0 to 34 months (M 5.8, *SD* 9.3). Only a minority (*N* = 5) had been given a diagnosis and these were related to anxiety, depression, autistic spectrum disorder and attention deficit and hyperactivity disorder. Full demographic details can be seen in Table 1.

**Table 1.** Demographic information.

Variable	Participants
**Age** *Mean*(*SD*)	15(1)
**Age of voice hearing onset** *Mean*(*SD*)	12(4.2)
**Duration of voice hearing in years** *Mean*(*SD*)	3(4.1)
Gender n(%)	
Female	16(67)
Male	7(29)
Used another term	1(4)
Ethnic group n(%)	
White British	20(83)
White other/mixed ethnicity	3(13)
First language n(%)	
English	22(92)
Portuguese	1(4)
Highest educational attainment n(%)	
Left school before 16	1(4)
Still in school	15(63)
Completed/completing college	8(33)

*Note*. Percentages are based on all available data for the variable; data missing for characteristic: age of voice hearing onset (*n* = 1), duration of voice hearing (*n* = 1), ethnic group (*n* = 1), first language (*n* = 1).

### Baseline clinical characteristics

Data were available at baseline for all 24 young people on the primary outcome and high levels of negative impact of voices were reported on the HPSVQ (*M* 11.3, *SD* 2.5, range 8–16). On the secondary outcomes, young people reported high scores on the HPSVQ Physical scale (*M* 12.7, *SD* 2.9, range 7–20), ‘severe’ levels of anxiety on the GAD-7 (*M* 16.5, *SD* 3.79, range 8–21), ‘clinical’ levels of depression on the PHQ-9 (*M* 18.4, *SD* 6.0, range 5–26) and low ratings for recovery on CHOICE-SF severity (*M* 3.9, *SD* 1.14, range 2–7). Twenty young people created a CHOICE-SF goal prior to the commencement of the intervention. Goals were related to voice hearing experiences (*n* = 9), emotional wellbeing/mental health (*n* = 5) and self-improvement/personal growth (*n* = 6).

### Engagement

Five young people withdrew from SVC prior to the completion of the intervention (having attended between one and three sessions). Reasons for withdrawal were the following: a review of diagnosis suggesting that the intervention may not be suitable; changing domestic circumstances; cessation of voice hearing experiences for two young people; and not known. At the time of this service evaluation, four young people were waiting to start brief CSE. The remaining 15 young people completed the intervention, of whom 13 completed a post-intervention assessment. The reasons for non-attendance at the post-intervention assessment were a family emergency and ambivalence due to ‘not getting much’ from the intervention.

### Outcomes

The outcomes for the 13 young who completed the brief CSE intervention and offered full datasets are contained in Table 2. Our primary analysis demonstrated improvement on the HPSVQ negative impact of voices scale post-intervention when compared with the baseline score. The mean change post-intervention was −3.50 (95% CI: −6.40, −.68). This was a large standardised effect size of d = −0.96 and 10 young people reported a reduction that was equivalent to, or greater than, the MCID of 2 points. The results also indicated promising levels of improvement on the secondary outcome measure of recovery with large effects for the CHOICE-SF severity score (1.08, 95% CI 0.21, 1.96; d = 1.04) and the single item CHOICE-SF goal rating (3.8, 95% CI 2.48, 5.18; d = 1.70). Other secondary outcomes generated medium effect sizes for the physical characteristics of voices (mean reduction in HPSVQ physical scale scores of −2.36 [95% CI −5.71, 0.99; d = −0.54]) and depression (mean reduction in the PHQ-9 score of −3.58 [95% CI −8.94, 1.77; d = −0.63]), and a small effect size for anxiety (mean reduction in the GAD-7 score of −1.67 [95% CI −5.19,1.86; d = −0.34]).

**Table 2.** Summary of outcomes for young people who completed the CSE intervention and offered full datasets (*N* = 13).

Measure	Pre-intervention			Post-intervention			Effect size
	M	*SD*	Range	M	*SD*	Range	d
HPSVQ negative impact scale	10.54	2.18	8–14	7.00	4.58	0–16	0.96
HPSVQ physical scale	12.73	2.76	7–17	10.36	5.22	0–18	0.54
PHQ-9	18.92	4.60	9–25	15.33	6.62	3–26	0.63
GAD-7	16.58	4.06	8–21	14.92	5.55	7–21	0.34
CHOICE severity	3.75	.87	2–5	4.83	1.19	3–7	−1.04
CHOICE goal rating	3.00	1.35	1–6	6.83	2.55	2–10	−1.70

*Note*. Results are based on all available data for the variable; data missing for measure: HPSVQ Physical (*n* = 2), PHQ-9 (*n* = 1), GAD-7 (*n* = 1), CHOICE severity (*n* = 1), CHOICE goal (*n* = 1). HPSVQ = Hamilton Program for Voices Questionnaire; GAD-7 = generalised anxiety disorder assessment; PHQ-9 = patient health questionnaire; CHOICE = choice of outcome in CBT for psychoses – short form; CSE = coping strategy enhancement.

### Qualitative feedback

The young people offered feedback on several topics. Many talked about positive aspects of the intervention, for example, ‘I’m extremely satisfied with the therapy, it has helped a lot’. Six young people mentioned coping strategies and techniques, for example, ‘coping strategies and distraction techniques were helpful’, ‘it taught me lots of different ways to block out the voice, move forward and not dwell on things' and ‘I have learnt more ways to cope with the voices and how to cope with them in a positive way’. Seven young people discussed the value of the therapist and the therapy space. They talked about being ‘unsure about speaking to [the therapist]’ at first, and ‘getting to know the therapist a bit more before asking questions’. One young person said they have felt judged in the past when talking about their voices, but their therapist ‘was easy to talk to and just listened to me without judging’; similarly, others said the therapy was a ‘safe space’ and they felt ‘listened to’ and ‘able to talk to someone who would understand’. Finally, four young people said they would have liked to have had more sessions: ‘it ended a bit too soon’ and was ‘too short’.

## Discussion

Voice hearing experiences are common amongst young people, but there are currently no evidence-based interventions for treating the distress that can sometimes be caused by these experiences. Consistent with recommendations for a light-touch intervention (Maijer et al., 2019), we offered a brief form of CSE to young people who were distressed by hearing voices within the routine clinical environment of a Child and Adolescent Mental Health Service within the NHS. The level of engagement, outcomes and reported experiences offered encouragement and suggested that this brief intervention was acceptable and helpful to the majority of the young people.

Within this service evaluation, the cohort of young people were predominantly female, within the 14–16 age range, attending school and had not been diagnosed with a mental health condition. The age of onset and duration of their voice hearing experiences varied considerably. The clinical characteristics of the young people in this study were comparable to those reported by the adult patients of SVC, as they reported high levels of negative impact of voices and low levels of recovery (Clarke et al., 2021). One notable difference was the cessation of voice hearing experiences; this is unusual for adult patients but was reported by two of the young people within this service evaluation. This finding corroborates the suggestion that voice hearing can be a transient experience for young people (Bartels-Velthuis et al., 2016).

The levels of engagement with the brief CSE intervention were encouraging, with the majority (15 of 20) of the young people completing all four sessions. This finding suggests that the intervention was acceptable to the young people, and acceptability was corroborated by some of the comments within the qualitative feedback. In the context of some of the barriers to talking about voice hearing experiences for young people (Bogen-Johnston et al., 2019), the acceptability of this opportunity to talk about voices is noteworthy.

The findings regarding outcomes were similarly encouraging, as most of the young people who completed the brief CSE intervention and offered full datasets experienced a clinically meaningful reduction on the primary outcome of the negative impact of voices. The large effect size for this reduction compares favourably with the small-medium reduction reported for adult patients who completed brief CSE within SVC (Clarke et al., 2021). Despite experiencing reductions in negative impact that were clinically meaningful, a minority of young people (*N* = 4) continued to experience a high level of negative impact after the intervention (scoring 8 or more on the HPSVQ negative impact of voices scale), suggesting that further interventions to target their voice hearing experiences may have been needed. Mechanisms that could be targeted by further interventions may include negative beliefs about self and relational factors (Parry & Varese, 2021). Within SVC, options for further interventions are discussed with the young person and their care coordinator in the context of the young person’s care plan. Consistent with the primary focus upon reducing the negative impact of voices rather than attempting to change any characteristics of the voice hearing experience itself (Birchwood & Trower, 2006), there was only a medium effect size for the reduction on the physical characteristics scale of the HPSVQ.

Amongst the secondary outcomes, benefits were most noteworthy for the measure of recovery. Large effects were found for both the severity rating and for the personal goal. As was the case with the primary outcome, these effect sizes were larger than those reported for adult patients within SVC (Hayward et al., 2018).

This service evaluation has limitations in several respects. Firstly, the sample size was small and drawn from only a single CAMHS service. Consequently, there was insufficient statistical power to test for the significance of pre-post differences and findings may not be generalisable. Secondly, some of the outcomes measures used had been developed with adults and may not have been sensitive to the needs and experiences of young people. Thirdly, there was no control condition and the reductions in outcomes may have occurred naturally over time. Fourthly, the post-intervention assessments could not be conducted blind and may have been influenced by the assessor. Fifthly, there was no attempt to collect follow-up data and the durability of outcomes remains unknown. Finally, the participants lacked ethnic diversity and the acceptability and usefulness of the intervention for young people from a more diverse range of ethnic and cultural backgrounds requires exploration. Many of these limitations could be addressed by the conducting of a sufficiently powered multi-site randomised controlled trial, with blind assessments and collection of data at follow-up.

The findings from this service evaluation suggest that CSE can be a brief, acceptable and helpful way for young people within a CAMHS context to start a therapeutic conversation about their distressing voice hearing experiences. For some young people, a continuing high level of negative impact of voices post-intervention may require this conversation to be continued as part of a stepped approach to intervention for distressing voices.
